# Squamous cell carcinoma arising from a keratocystic odontogenic tumor: a case report

**DOI:** 10.1186/s13256-017-1486-x

**Published:** 2017-12-01

**Authors:** Rajapakse Mudiyanselage Sumudu Himesha Bandara Medawela, Nadeena Sri Swarnaguptha Jayasuriya, Dugganna Ralalage Dilini Lalanthi Ratnayake, Ajith Manjula Attygalla, Bogahawatte Samarakoon Mudiyanselage Samadarani Siriwardena

**Affiliations:** 1 Department of Oral Medicine and Periodontology, Faculty of Dental Sciences, University of Peradeniya, Peradeniya, Sri Lanka; 2 Department of Oral and Maxillofacial Surgery, Faculty of Dental Sciences, University of Peradeniya, Peradeniya, Sri Lanka; 3 Department of Oral Pathology, Faculty of Dental Sciences, University of Peradeniya, Peradeniya, Sri Lanka

**Keywords:** Primary intraosseous squamous cell carcinoma, Keratocystic odontogenic tumor, Malignant transformation

## Abstract

**Background:**

The term “primary intraosseous squamous cell carcinoma” was introduced in 2005 by the World Health Organization with three subcategories. Squamous cell carcinoma arising from the lining of an odontogenic cyst is one important rare subcategory of such lesions with an incidence of 0.01 to 0.02%. Furthermore, the appearance of such malignancy in an odontogenic tumor such as keratocystic odontogenic tumor is considered extremely rare.

**Case presentation:**

In this case report we report a case of a 50-year-old Sri Lankan woman who complained of pain and increase in the size of a swelling at the anterior mandible, which had been present for over 1 year. The increase was significant for 1 month with accompanying numbness of the left half of her lip. Cone beam computed tomography results revealed an irregular radiolucent lesion involving most of her mandible and, except in the anterior part, very little buccolingual expansion was seen that suggested a keratocystic odontogenic tumor. An excision biopsy of the cyst lining confirmed a squamous cell carcinoma arising from a preexisting keratocystic odontogenic tumor.

**Conclusions:**

Even though primary intraosseous squamous cell carcinoma arising from a keratocystic odontogenic tumor is considered to be very rare, the present case is comparable to most of the aspects cited in the literature. The current case emphasizes the importance of careful investigation of swellings present in the mandible. Clinicians as well as patients should be aware and detect these changes to avoid being clinically negligent.

## Background

Primary squamous cell carcinoma (SCC) arising within jaw bones with an odontogenic origin is termed primary intraosseous odontogenic carcinoma (PIOC). Intraosseous variants of SCC appearing in maxillary and mandibular regions are rare and the rate of incidence is unknown [1, 2].

A literature review on the evolution of terminology of such lesions has revealed that similar pathological phenomena were termed “central epidermoid carcinoma” by Loos in 1913, “intraalveolar epidermoid carcinoma” by Willis in 1948, and “primary intraalveolar epidermoid carcinoma” by Shear in 1969 [1]. The term PIOC was suggested by the World Health Organization (WHO) in 1972. However, the initial classification of PIOC was modified taking to account its tissues of origin [2].

The term “primary intraosseous squamous cell carcinoma” was introduced in 2005 by the World Health Organization with three subcategories: type I for solid tumors, type II for carcinomas arising from odontogenic cysts, and type III for carcinomas associated with odontogenic tumors. With modifications, the WHO in 2005 classified PIOSCC into three subcategories as follows [1]:

I. Solid tumors that invade marrow spaces and induce osseous reabsorption.
II. SCC arising from the lining of an odontogenic cyst: this subdivision includes carcinomas arising in keratocystic odontogenic tumor (KCOT) and carcinomas arising from other odontogenic cysts.
III. SCC in association with other benign epithelial odontogenic tumors.

PIOSCC arising from a KCOT is rare. Bodner and colleagues’ [2] review of 116 cases of primary SCCs derived from odontogenic cysts revealed an incidence of 0.01 to 0.02% for origin from KCOTs [1–4]. Therefore, the treatment response and prognosis of such lesions are not known. We present the case of a SCC (T4N0M0) in a long-standing KCOT of the anterior mandible, which showed good response to surgical treatment. The current case is the first PIOSCC arising from KCOT reported in Sri Lanka.

## Case presentation

**Fig. 1:**
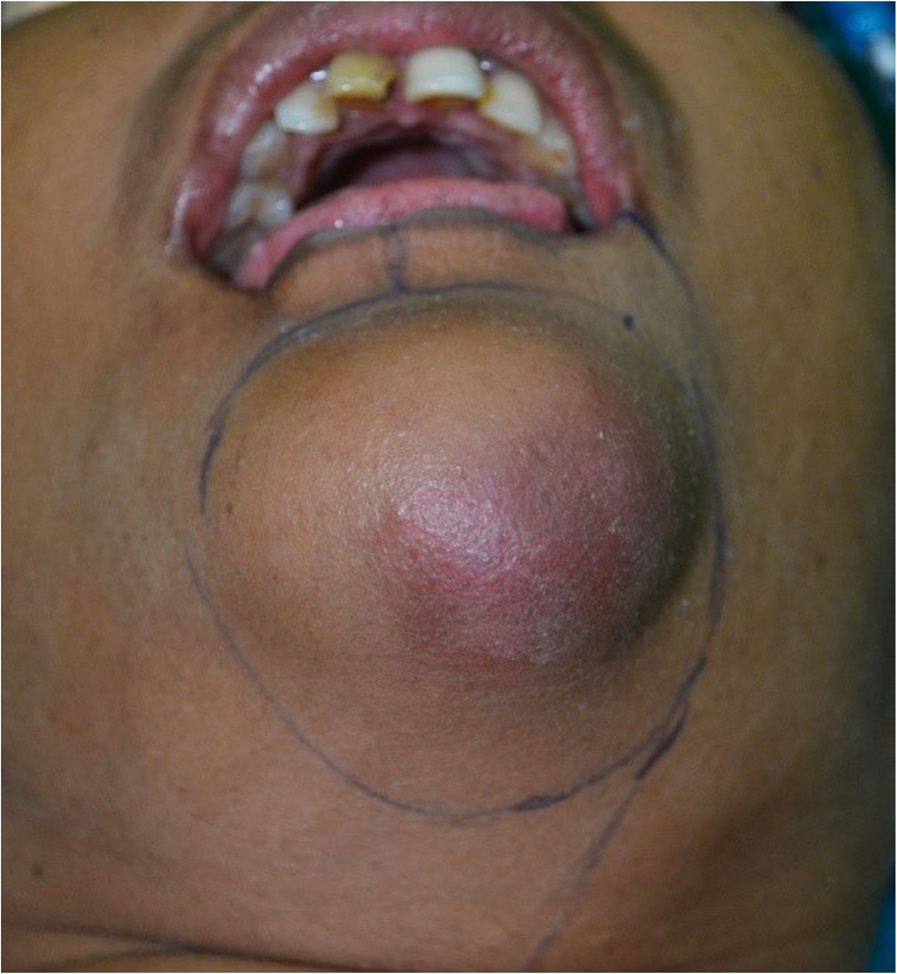
Swelling on the mandible

A 50-year-old Sri Lankan woman complained of pain and increase in the size of a swelling on her anterior mandible, which had been present for over 1 year (Fig. 1). This significant change in size was noted 1 month prior to presentation with accompanying numbness of the left half of her lip. She was otherwise healthy (not on any medication) and her past medical and social history (she did not have risk habits such as tobacco smoking, betel chewing, smokeless tobacco, and alcohol) did not reveal any significant contributions to her current presentation. She was a homemaker and had two children. Exploration of her family history did not reveal that a similar pathology affected her parents, siblings, and children.

A general examination of our patient revealed no significant findings and all vital signs (blood pressure, pulse rate, temperature, and respiratory rate) were within normal range. A well-defined, 4 × 4 cm swelling with redness on the overlying skin was noted on her submental region towards the left side. On intraoral examination, extensions of the same swelling measured 2 × 1.2 cm in size and involved the 31, 32, 41, and 42 teeth. No skin involvement was noted and the swelling appeared to be ill defined. The swelling was bony hard in consistency. Neurosensory evaluation revealed loss of sensation for fine touch, pressure, and temperature in lower left-sided labial skin.

**Fig. 2:**
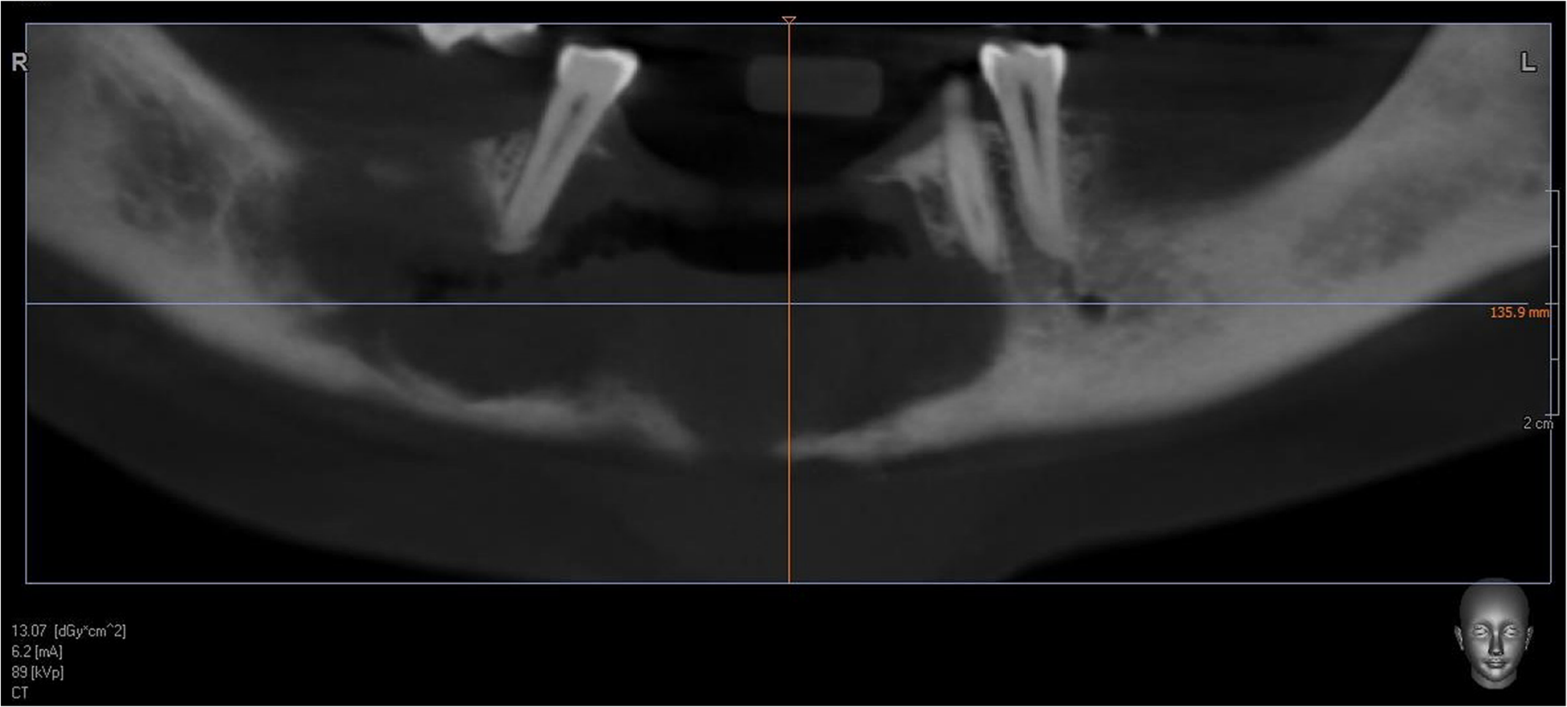
Cone beam computed tomography of the mandible. Pseudo-dental pantomogram view of lesion

**Fig. 3:**
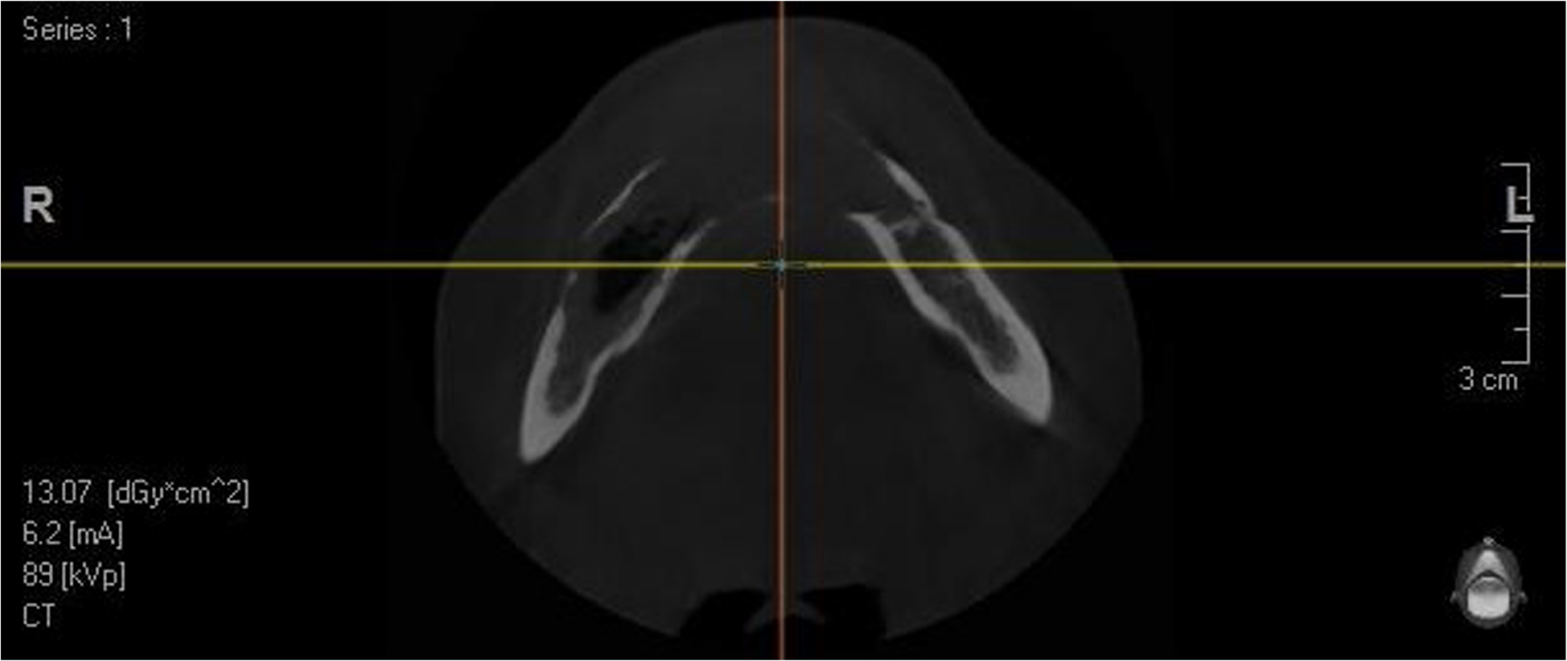
Cone beam computed tomography of the mandible. Axial view of lesion

These changes of recent onset were suspected to be an infection of an odontogenic cyst of her mandible and a cone beam computed tomography (CT) was performed. The results showed an irregular radiolucent lesion involving most of her mandible from the 35 to the 47 region. In the anterior part, both buccal and lingual cortical bone erosion was seen from the 33 to the 44 teeth of the mandible (Figs. 2 and 3). Except in the anterior part, very little buccolingual expansion was seen that suggested a KCOT. The roots of the 34 and the 44 teeth were involved in the lesion but no root reabsorption was evident.

Routine hematological investigations were carried out (full blood count, serum electrolytes, and liver function test) with renal function test and the results were well within limits.

An incisional biopsy revealed a SCC arising from a preexisting KCOT. An ultrasound scan and a CT scan of the bilateral neck revealed no metastasis migration to the neck nodes (T4N0M0).

Following incision biopsy, left-side neck dissection (levels I to III) and segmental mandibulectomy to include a bony and a soft tissue margin of 1 cm were performed. The bony and soft tissue defects were reconstructed with a titanium reconstruction plate and a pectoralis major myocutaneous flap. Her recovery was uneventful. She had mild discomfort during the first few months following surgery and adapted to the changes subsequently. Close to the 16 months’ review, the titanium plate was seen externalizing intraorally. She complained of a mild pain during wide opening of her mouth due to the reconstruction plate. She had been routinely reviewed in the clinic with 1-month review intervals and currently she has been disease free for the last 18 months; a free fibula flap is planned for the mandibular reconstruction.

**Fig. 4:**
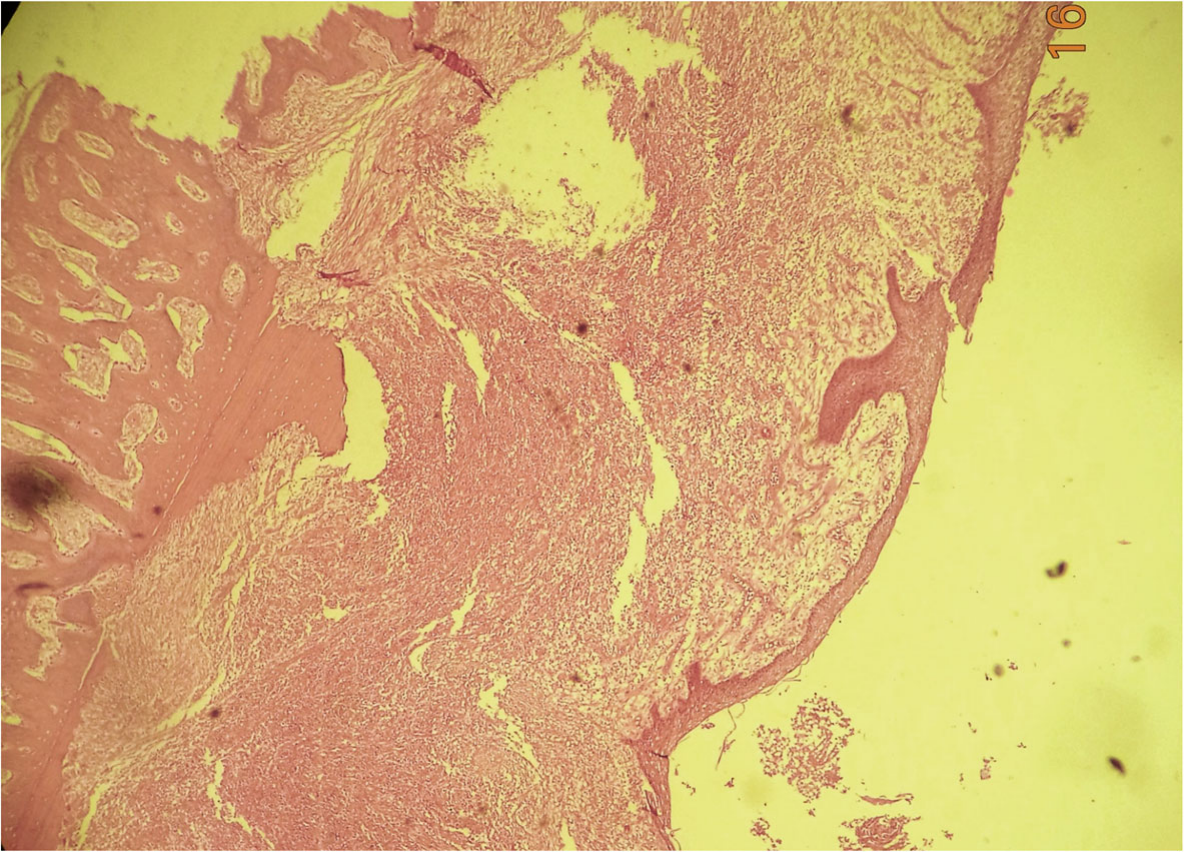
Histopathological section of keratocystic odontogenic tumor

**Fig. 5:**
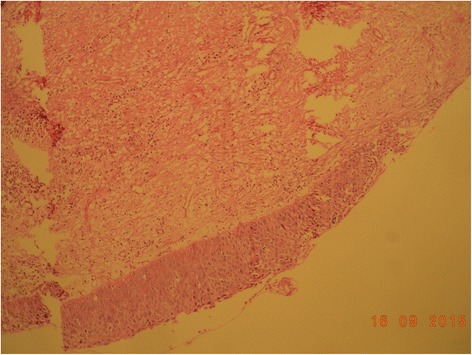
Epithelial lining of keratocystic odontogenic tumor at higher magnification

**Fig. 6:**
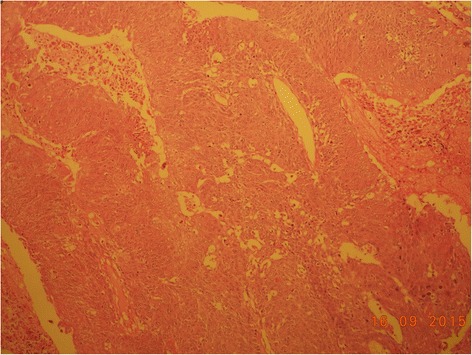
Squamous cell carcinoma appearing in keratocystic odontogenic tumor

On histopathologic examination the lesion was reported as a moderately differentiated SCC arising from a KCOT (Figs. 4, 5, and 6). Complete excision of the lesion was reported in the biopsy and the left-side neck nodes of all three levels were negative.

**Fig. 7:**
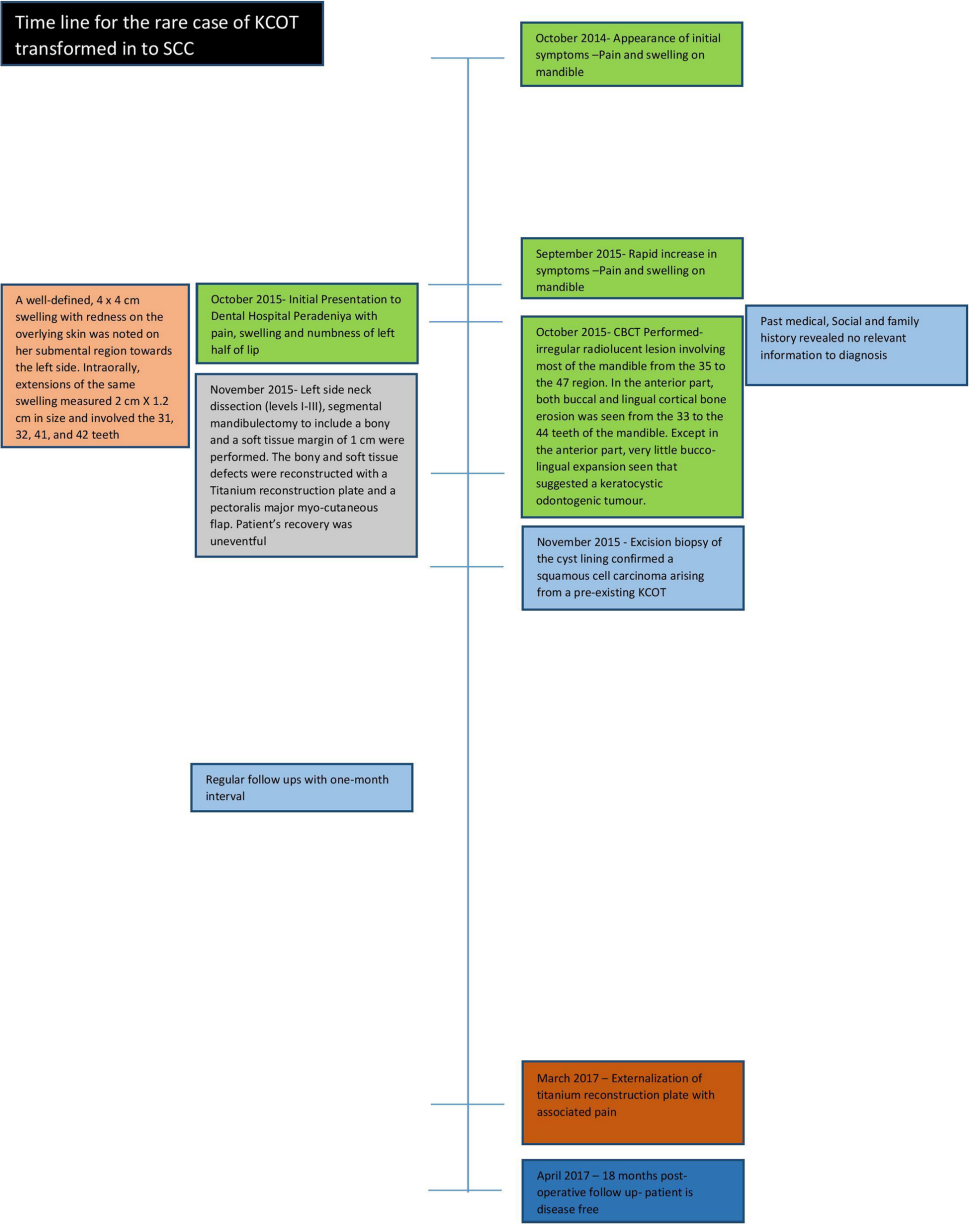
Timeline. cm - Centimeters, KCOT - Keratocystic odontogenic tumour, SCC - Squamous cell carcinoma

Postoperative radiotherapy or chemotherapy was not prescribed considering the histopathology findings (a timeline is shown in Fig. 7).

## Discussion

The pathogenesis of PIOSCC arising from KCOT had been debatable and several mechanisms were suggested by different pathologists [1]. Browne and Gouch highlighted a few important landmarks in the pathogenic sequence such as epithelial hyperplasia, epithelial dysplasia of cyst epithelia, and finally keratin metaplasia [5]. In consideration of the above landmarks in pathogenesis, van der Wal *et al. *mentioned that the presence of keratinization in the cyst lining results in the development of SCC in odontogenic keratocysts [6]. Gardner *et al.* and Yu *et al.* viewed this from a different perspective and suggested that the formation of reactive oxygen metabolites in long-standing inflammation resulting in damage to the dioxy ribonucleic acid (DNA), protein, and cell membranes eventually leads to compensatory proliferative responses of neoplastic cells against the normal apoptotic mechanism [7, 8].

Bodner* et al. *[2] in 2011 reviewed the literature on PIOSCC in odontogenic cysts and Table 1 elaborates a comparison of their data with the present case.

**Table 1:** Comparison between Bodner and colleagues’ [2] analysis of 116 reported cases of squamous cell carcinoma arising in an odontogenic cyst and the present case

Feature	Literature review		Present case	Comment
Age	Mean age	60.2 years	50 years	Close to mean age
	Commonest age of occurrence	6–8th decades		
Gender	Male	68%	Female	Male predilection
	Female	36%		
Site	Mandible	79%	Mandible	
	Maxilla	21%		
Type of cyst	Radicular cyst	60%	KCOT	KCOT malignant transformation rate is rare
	Dentigerous cyst	16%		
	KCOT	14%		
	Lateral periodontal	1%		
	Unclassified	9%		
Histopathologically	Carcinoma *in situ*	3%	Moderately differentiated SCC	
	Well-differentiated SCC	45%		
	Moderately differentiated SCC	40%		
	Poorly differentiated SCC	7%		
	Verrucous carcinoma	3%		
	Spindle cell carcinoma	1%		
Signs and symptoms	Asymptomatic	11%	Mass paresthesia present in the lower lip	
	Mass	32%		
	Pain	24%		
	Painful mass	16%		
	Jaw expansion	14%		
	Sensory disturbance	3%		
Treatment modality	Surgery alone	46%	Surgery with neck dissection	
	Surgery with radiotherapy	38%		
	Surgery, radiotherapy with chemotherapy	6%		
	Surgery with chemotherapy	6%		
	Radiotherapy alone	4%		
	Neck dissection	51%		
Overall survival	2 years	62%	1.5 years	Patient still under review
	5 years	28%		

*KCOT* keratocystic odontogenic tumor, *SCC* squamous cell carcinoma

In their study Bodner* et al. *highlighted the presence of only 16 cases of PIOSCC arising from KCOT from 1938 to 2010 [2]. Further, there were two other cases reported in the literature by Lee *et al.* [9] in 2011 and Tamgadge *et al. *[1] in 2013. A review of the literature regarding PIOSCC showed the mandible (79%) as the predominant site of occurrence as compared with the maxilla (21%). Similar data were recorded in the present case as the lesion appeared in the mandible. In accordance with the literature, PIOSCC arising from a KCOT shows a wide age range, with a mean age of 57 years, and males outnumbered females with male–female ratio of 2:1. Similar findings were observed in studies conducted by Aboul-hosn Centenero *et al.*, Mosqueda-Taylor *et al.*, and Scheer *et al.*; however, our patient was a 50-year-old woman.

Even though SCC arising from KCOT is a rare phenomenon, the present case is comparable with most of the aspects cited in the literature with the exception of our case being female. Also, PIOSCC arising from KCOT is considered to be very rare whereas a radicular cyst is more common.

Some reported cases of PIOSCC demonstrated symptoms of pain, progressive swelling, and paresthesia at the initial stages of the disease. Paresthesia was associated with the current case and the pain that appeared in the latter stage was the trigger for the patient to present to the healthcare facility. Such avoidance leads to delayed presentation and poor prognosis of oral malignant lesions in the South Asian population.

Data on the prognosis of PIOSCC arising from KCOT is scarce as there are only a few reported cases. However, in the reported cases, the 2-year survival rate of patients has been 53%. The prognosis was reported to be poor when there was evidence of nodal metastasis of the neck [10]. Surgical management was recommended in most reports with adjuvant radiotherapy [11]. Radiotherapy was not given to this current case because she was tumor free and adequate resection margins were confirmed in the excision biopsy. Furthermore, no neck nodal metastases were observed histopathologically. Neck metastasis is considered to be an important prognostic indicator and according to Bodner et al. only six cases of PIOSCC arising from odontogenic cysts have been reported [2].

Debates exist about the features of the grading of a tumor among different pathologists regarding grading of tumors because it is subjective. Therefore, the data to consider prognosis for each category of grading is unreliable.

According to the current case, we emphasize the importance of careful investigation of swellings present in the mandible. Clinicians as well as patients should be aware and detect these changes to avoid being clinically negligent. Careful histopathological examination of an odontogenic cyst is also recommended as, even though it is rare, there is a possibility for its epithelial lining to become malignant.

## Conclusions

PIOSCC arising from KCOT is an unusual and rare malignant lesion of the maxillofacial region and this article reviewed the case of a 50-year-old woman who presented with the most typical features of such a presentation. This case report highlights the importance of thorough investigation of each complaint to improve prognosis.
